# An exploration of cultural influencing factors on dietary diversity in Malagasy children aged 6–59 months

**DOI:** 10.1186/s40795-022-00509-8

**Published:** 2022-03-11

**Authors:** Jacqueline Ribeli, Franziska Pfister

**Affiliations:** 1 School of Agricultural, Forest and Food Sciences HAFL, Bern University of Applied Sciences, Länggasse 85, 3052 Zollikofen, Switzerland; 2 Health Department – Nutrition and Dietetics, Bern University of Applied Sciences, Murtenstrasse 10, 3008 Bern, Switzerland

**Keywords:** Child nutrition, Malnutrition, Dietary diversity, Stunting, Cultural habits, Madagascar

## Abstract

**Background:**

More than 1.7 million children in Madagascar are stunted, with low dietary diversity being a determinant. Although diverse crops are produced in the Vakinankaratra region, it registers the highest stunting prevalence rate nationally. While children’s diets may be influenced by region-specific cultural factors, little is known about this link so far. The aim of this study was to determine the influence of cultural habits on dietary diversity in children between 6 to 59 months in the Vakinankaratra region.

**Methods:**

A mixed method design with a qualitative lead approach was used, including three transect walks, six focus group discussions and 51 semi-structured interviews with caregivers. The interviews contained a quantitative part to assess the four feeding indicators: ‘minimum meal frequency’, ‘minimum dietary diversity’, ‘minimum acceptable diet’ and the consumption of iron-rich foods. Participants were selected by convenience sampling. Data was collected in November 2019 in three communities displaying maximal possible variation.

**Results:**

Subsistence farming with diverse crops and livestock was found to be a common practice. Minimum meal frequency was achieved by 78% of the sixty included children. In total, 45% attained minimum dietary diversity and 40% consumed a minimum acceptable diet. Across the three communities, the range of children attaining a minimum acceptable diet was 25–60%. Older, no longer breastfed children were prone to not achieving a minimum acceptable diet. Although caregivers had a basic idea of important foods for child development, these were often not available to or affordable for them. An effect of cultural events on diet, especially diversity, was found. Whilst for annual events this effect was short-term, the region-specific ceremony of reburying the dead (Famadihana) was found to have a long-term influence on the diet of certain families.

**Conclusions:**

The dietary diversity of children under five in the Vakinankaratra region is influenced by cultural factors like agricultural practices, caregivers’ knowledge of child nutrition, food taboos and a region-specific cultural event. Cultural determinants, especially important traditions that result in significant expenditures, may thus influence the quality of children’s diet and should therefore be considered in future nutrition programmes and research on child malnutrition and stunting.

## Background

Stunting denotes growth failure in children, making them too short for their age [1]. The consequences for children go far beyond not reaching their full potential height and can last throughout the lifecycle [2]. In the short-term, stunting is associated with greater morbidity and mortality due to infections, especially diarrhea and pneumonia [3, 4]. In the long term, stunted women tend to give birth to stunted offspring. This intergenerational cyclical process is difficult to break, and perpetuates the poverty cycle [2, 5]. As linear growth is not assessed in all primary health centers, stunting often remains unrecognized in communities where a shorter height is considered normal. This makes it even more difficult to identify and combat stunting [6].

Madagascar is one of the poorest countries in the world [7]. Globally it has one of the highest stunting rates, with more than 1′700’000 children below the age of five being affected, with the highest prevalence in the Vakinankaratra region (60%) [8]. The region is located near the capital and hosts mainly farming households [9]. Low maternal height is an underlying predictor of child stunting [10]; around 45% of mothers are shorter than 150 cm in the region [11]. In Malagasy studies, children perceived by their mothers to be small at birth were shown to be at increased risk of becoming stunted [10, 12, 13]. Rakotomanana et al. (2017) examined which infant and young child feeding practices indicators were associated with stunting in Madagascar. They found a higher average length-for-age in children attaining minimum dietary diversity (MDD) [14]. Low maternal education was associated with a high chance of inadequate dietary diversity in children [15]. Poor nutritional quality was mainly due to a carbohydrate-rich diet containing inadequate amounts of animal products [16]. The risk of stunting increased with age [10, 13] being particularly associated with the 12–35 months age range [12]. Furthermore, the risk of stunting increased in the presence of severe or moderate anemia in children below the age of two, whereas in the older group of 24–59 months of age, a mild to moderate anemia sufficed to increase the risk [10]. Despite decades of projects fighting malnutrition in Madagascar, there has been little improvement in stunting rates [8, 17].

Caregivers’ beliefs around child growth, malnutrition and health are culturally bound [18, 19]. Cultural constructs affect complementary feeding practices. Interventions for the prevention of childhood malnutrition often address biological determinants. An alignment with the cultural models and habits of the community in question is required to be effective [19]. The concept of culture in this context needs to be broad, including ordinary aspects of everyday life [20], e.g. dietary habits, the value attributed to different foods, and crop diversity and marketing in a rural setting.

Even though many determinants of stunting in Madagascar have been identified, little is known about important cultural influences. Thus, the following overall research question was developed: How do cultural habits influence dietary diversity of children between 6 and 59 months of age in the Vakinankaratra region of the highlands of Madagascar? As a secondary aim, data on infant and young child feeding practices was collected.

## Methods

A mixed-method approach was used in this study. Qualitative data was collected with three methods: Transect walks, focus group discussions (FGD), and semi-structured interviews. Quantitative data was additionally gathered to assess children’s dietary quality. These methods were combined to obtain more diverse and complementary results, via different emphases in the questionnaires as well as altered conversation settings, and to allow for triangulation of information.

All data was collected in November 2019 by a research team comprising of the Swiss dietitian JR, the lead researcher, and two Malagasy physicians with work experience in the study region. The latter served as local research partners and translators. Questions were asked in English and translated into Malagasy. Answers were translated from Malagasy to English. All conversations were voice recorded.

The interview guides for each method were based on an operationalization table, derived from the main research question. A sociodemographic questionnaire adapted from Fautsch Macías and Glasauer (2014) [21] was carried out with all the participants. It included information on gender, age, relationship to child, parity, educational level, and gender and age of the children referred to in the interviews or the FGDs.

### The transect walks

based on the example of Selener, Endera and Carvajal (1999) [22], aimed at developing an understanding of the local farming practices, the marketing or consumption of the produce, and the cultural value attributed to the different crops. Furthermore they allowed for a deeper understanding of the respective community. One transect walk was carried out per community, always with a farm household head, who had been living in the respective community for several years and owned at least one plot. The conversation followed a semi-structured interview guide with the following questions: 1. What foods are currently produced? 2. How are the fields managed? 3. What happens with the harvested food? 4. Which crops or animals are sold, which are kept and why? 5. What is the most valuable food to sell? 6. What is the money used for? 7. Which of the produced foods is used for feeding the family? 8. What is the specific role of rice? The transect walks lasted about one and a half hours.

### Focus group discussions

aimed at analyzing specific cultural habits that affect child development and diet. The group setting is particularly suitable in communities with a low level of literacy and an oral tradition. It facilitates to the answering of potentially delicate questions (e.g. food taboos, financial constraints). Two FGDs with four to seven mothers or grandmothers caring for (grand) children below the age of five were carried out in each community, as literature shows that in two to three FGDs more than 80% of themes are discoverable [23]. After the entry question, the semi-structured questionnaire focused on the following topics: 1. Knowledge on child-specific nutrition; 2. Influencing factors and decision-making power about children’s diet; 3. Food taboos; 4. Financial resources and special events. The FGDs were co-moderated by JR and a local research staff and lasted about 45 min. Questions and answers were translated constantly. The FGDs took place outdoors, with participants and moderators sitting on the ground.

### Semi-structured face-to-face interviews

with caregivers in charge of at least one child between the age of six and 59 months were chosen as the main data collection tool. We aimed at a minimum of seven interviews per community for qualitative data, as even a lower number of interviews allows for data saturation in qualitative research [24]. They focused on children’s diets, food consumption from homegrown produce and cultural influencing factors. The interviews were carried out in different settings, mostly at participants’ homes, and lasted between 10 and 50 min.

In the second part of the interviews, data for the World Health Organization (WHO) infant and young child feeding (IYCF) indicators were collected. These assess the nutritional quality of diets for children between the ages of 6–23 months [25, 26]. In this study, they were also used for older children (24–59 months), as this age group is at a high risk for stunting in Madagascar [12] and micronutrient malnutrition due to low dietary diversity is common in this age group in Sub-Saharan Africa (e.g. [27, 28]). The following four indicators were included: 1. Minimum meal frequency (MMF); 2. Minimum dietary diversity (MDD); 3. Minimum acceptable diet (MAD); 4. Consumption of iron-rich or iron-fortified food. MDD is especially useful for determining the quality of children’s diet in developing countries [29].

In this study, MMF was measured with an interactive tool: a timeline-image and magnets containing pictograms of breastfeeding, meals, and snacks. Caregivers indicated the child’s food consumption of the previous day by placing the corresponding magnets on the timeline. MMF was achieved when the following values were reached: a meal or snack twice a day for 6–8 month or three times a day for 9–23 month old, breastfed children, and four times a day for non-breastfed children in the 6–23 months age range [25]. As no validated recommendations were available for the older age group (24–59 months), the research team applied the same threshold values as for the oldest age group under 2 years – three meals when still breastfed, four when no longer breastfed. Due to small stomach size, three daily meals may not be enough to cover the energy requirements of a preschool-aged child [30, 31].

The seven food groups recommended by the WHO for MDD surveys on children under 2 years of age were included: 1. *Cereals, roots & tubers*; 2. *Vitamin A rich fruits & vegetables*; 3. *Other fruits & vegetables*; 4. *Legumes & nuts*; 5. *Flesh foods*; 6. *Dairy products*; 7. *Eggs*. One point was awarded for each food group consumed by the child, irregardless of the amount. The child attained MDD with a minimum of four points [25, 26]. As a supportive tool, the research team showed collages on posters for each food group during interviews. One collage for vitamin A-rich fruits and one for vegetables was created respectively, to capture any differences in vitamin A sources between communities. Nevertheless, they were treated as one food group when calculating the dietary diversity score. For each food group, interviewees were asked whether their child had eaten a food belonging to the respective group the day prior to the interview. The research team referred to the FAO guidelines when it was unclear which food group a certain food belonged to [32]. To assess a child’s consumption of iron-rich or iron–fortified foods, a collage with relevant foods was shown and specific supplements were asked for verbally.

Only children reaching both MMF and MDD were considered to have a MAD, as per WHO recommendation [26].

### Sampling

The Vakinankaratra region consists of six districts and two biogeographic zones, the highlands and the Middle West. There are major differences between the latter in terms of geography and the prevailing agricultural system [33]. Two districts in the highlands (Ambatolampy and Antsirabe II) and one in the Middle West area (Mandoto) were chosen. Dietary diversity, agricultural and cultural practices and beliefs may be influenced by the proximity of the next town, access to a local marketplace, economic structure and access to schooling. Therefore, one community in each district was selected together with the local staff, to ensure maximal variation in the dimensions mentioned above as far as possible. The choice was narrowed down by restrictions regarding access and security issues.

The study was conducted in the following three communities (Table 1): Behenjy is located on the national road, not far away from the capital. There is a marketplace that is open daily. Ibity is the most remote community of the sample, while Ankazomiriotra is between the two in terms of remoteness. Accordingly, the economic structure and access to schooling differs between the three communities (Table 1).

**Table 1:** Included communities and factors determining purposive sampling (based on [34])

**Community**	**District**	**Biogeographic zone**	**Economy**	**Schooling**	**Remoteness (1 = least remote; 5 = most remote)**
**Behenjy**	Ambatolampy	Highland	Agriculture, handicraft, industry and services	Primary and secondary schooling	1
**Ibity**	Antsirabe II	Highland	Agriculture (98%)	Only primary schooling	2
**Ankazomiriotra**	Mandoto	Middle West Area	Agriculture (75%), animal husbandry (25%)	Primary and secondary schooling	1

**Fig. 1:**
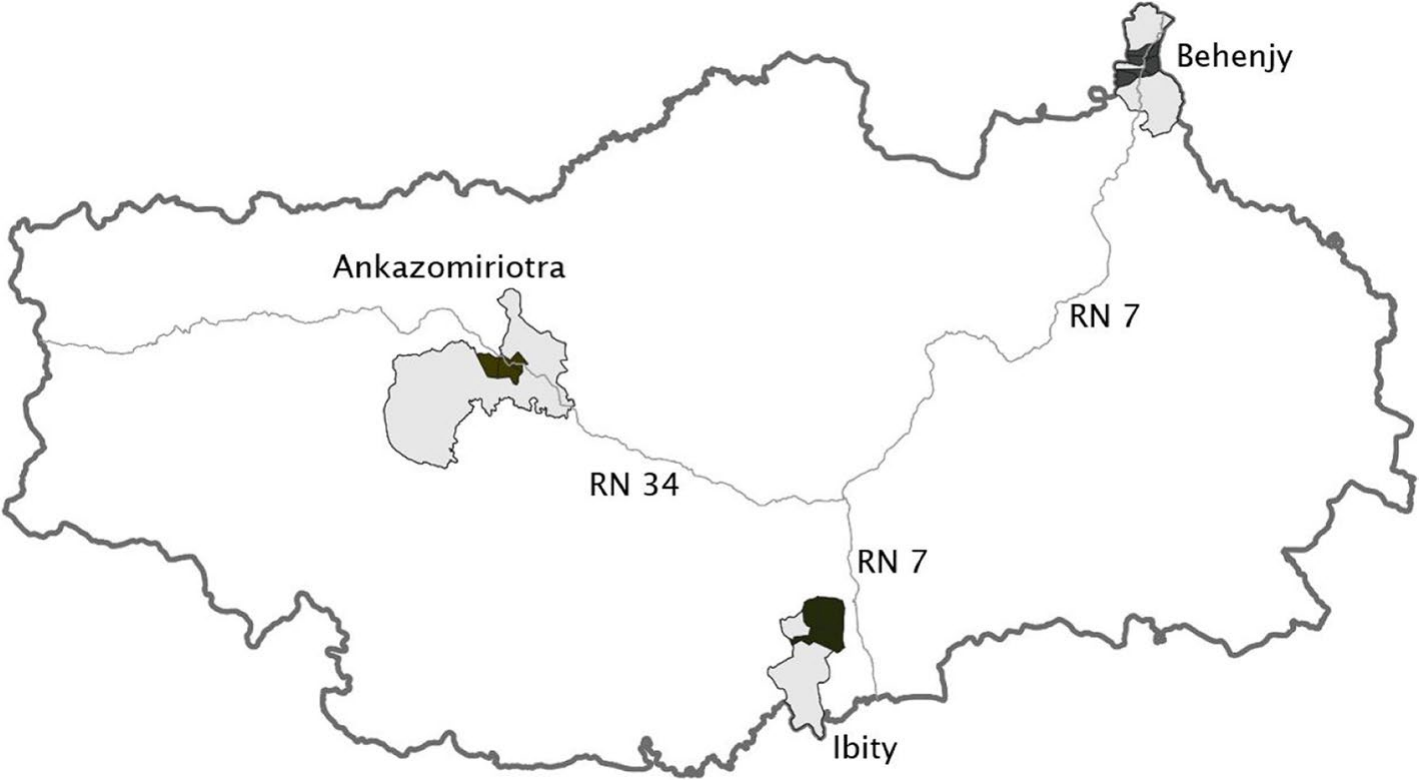
Communities - Behenjy, Ibity and Ankazomiriotra. Included communities and their fonkontany/villages. Study sites (fokontany) in black above: Behenjy: Marovato, Ambohikambana, Tsarafara, Soaloka, Mandady, Ambohidrano sud, Morarano (west). Ibity: Ambalavao, Ambarinakanga, Manajary. Ankazomiriotra: Ankazomiriotra I, Ankazomiriotra II, Andranovory. Lines delineate the main national roads: Route National (RN) number 7 and 34. Source: Adapted by Mirindra Rajoelison from Map in OSGeo4W-Software 2020

All participants for the transect walk, FGDs and interviews were selected by convenience sampling. The research team selected fokontany (villages) reachable by foot from its camp. They then walked around the fokontany and randomly asked potential participants whether they were willing to participate in the study, if they were found to fulfill the selection criteria. In some fokontany, the chief or health workers introduced the research team to women meeting the inclusion criteria. To include data from a variety of sources, FGDs were carried out in two different fokontany from each community (except in Ankazomiriotra), and several caregivers were interviewed from each included fokontany. Only people whose main residence was in the respective community were included. Figure 1 highlights the included study sites.

### Pretest

A pretest of the personal interview was carried out with a young mother of another Malagasy district. The pretest was analysed and questions were optimized to improve understandability. The interview guides for the FGDs and transect walks were not pretested, but all questions were checked for cultural appropriateness and understandability by a Malagasy nutrition researcher. The World Health Organization’s infant and young child feeding indicators are well established and widely used in similar settings.

### Characteristics of participants

A total of 60 conversations during 3 transect walks, 6 focus group discussions and 51 personal interviews were carried out. Transect walk and FGD participant characteristics are presented in Table 2. The characteristics of personal interviewees and their children included in the quantitative questionnaire are listed in Table 3. 20 children were included from each community.

**Table 2:** Characteristics of participants in research settings with qualitative data only

**Method**	**Characteristic**	**Behenjy**	**Ibity**	**Ankazomiriotra**
**Transect walk**	Participant (all males)	*n =* 1	*n =* 1	*n =* 1
	Age	49 years	63 years	27 years
	Educational level	Primary	Primary	None
**Focus group discussion 1**	Participants (all females)	n = 4	n = 7	n = 6
	Age, mean (range)	28.3 (20–45)	33 y (20–39)	28.2 y (20–50)
	Educational level	Prim.: 2/ Sec.:1/ NA: 1	Prim.: 5/ Sec.: 2	Prim.: 3/ Sec.: 3
**Focus group discussion 2**	Participants (all females)	*n =* 4	*n =* 4	*n =* 4
	Age, mean (range)	29.7 (23–34 /1x NA)	36.8 y (20–63)	23.8 y (19–32)
	Educational level	Prim.: 2/ High.: 1/ NA: 1	None: 1/ Prim.: 3	Prim.: 2/ Sec.: 2

**Table 3:** Characteristic of participants & children from semi-structured interviews

Characteristic	Behenjy	Ibity	Ankazomiriotra
**Participants**	*n* = 17	*n* = 15	*n* = 19
Mother/father/grandmother	16/0/1	15/0/0	18/1/0
Age, mean (range)	25.9 years (18–51)	25.7 years (18–43)	27.4 (19–45)
Number of children (mean/range)	1.9 (1–4)	2.7 (1–8)	2.4 (1–8)
**Education level**			
None**,** n (%)	0 (0%)	2 (13.3%)	2 (10.5%)
Primary, n (%)	8 (47.1%)	6 (40%)	9 (47.4%)
Secondary, n (%)	8 (47.1%)	4 (26.7%)	8 (42.1%)
Higher, n (%)	1 (5.9%)	3 (20%)	0 (0%)
**Included children**	*n* = 20	*n =* 20	*n =* 20
Age in months, mean (range)	27.9 m (7–58 m)	29.6 m (7–54 m)	32.1 m (7–59 m)
6–23 months (breastfed = Yes)	*n* = 9 (8)	*n* = 8 (8)	*n =* 6 (6)
> 23 months (breastfed = Yes)	*n* = 11 (0)	*n* = 12 (4)	*n* = 14 (3)

M = Method; TW = Transect Walk; FG = Focus Group Discussion; Prim. = Primary; Sec. = Secondary; High. = Higher.

Participants had a comparable age range. More than one child could be included from one participant. For the MMF indicator, children below the age of two were split into the age groups recommended by WHO (2008a) (6–11 months, 12–17 months, 18–23 months). Most other results were summarised in two age groups (6–23 months and >  23 months) due to the small sample size and the only minor differences within the WHO age groups. For the analysis of socio-economic factors, children were only examined by location and.

### Methodology of data analysis

For the quantitative data, a printed template including the four indicators was used directly during the interviews. All socio-economic information and quantitative data were transferred to Excel tables. Absolute and relative frequencies were analyzed and compared between communities and age groups (children between 6 and 23 months and 24–59 months) using RStudio software, version 1.1.463. Additionally, plots focusing on mean dietary diversity scores were created according to specific variables such as community, age group or child breastfeeding status. Due to the small sample size, no further statistical analyses were relevant for the quantitative data.

Qualitative data was thematically analyzed as described by Braun and Clarke (2013) [35]. The English translation of all voice-recorded data from all settings was transcribed with f4transkript v7 (students) software according to orthographical standard [36]. A period of familiarization with the transcripts followed, after which JR created the first deductive codes. With ATLAS.ti 8 software, version 8.4.22.0, additional inductive codes were created. All transcripts were fully coded across the entire dataset. Statements from all three qualitative settings were treated equally. Complete coding led to a total of 113 codes. Similar codes were grouped into themes [35]. Two overarching themes including six themes and eight additional subthemes emerged, as presented in Table 4 below. All themes were linked to different cultural topics related to children’s dietary diversity. Only the relevant themes are presented in this paper.

**Table 4:** Overview of created themes

Dietary diversity in Malagasy children aged 6–59 months		
Overarching themes	Themes	Subthemes
**1. Agricultural practices, consumption of homegrown produce & food procurement**	1.1 General aspects of crop production 1.2 Animal husbandry 1.3 Value and selling price of animal- and plant-based products	1.1.1 Activity calendar (transect walk) 1.1.2 Aspects around planting (input) 1.1.3 Use of harvested food 1.2.1 Role and usage of animals and their products
**2. Cultural - & socioeconomic aspects around food, people & events**	2.1 Family culture 2.2 Caregivers’ knowledge and perceptions of child nutrition 2.3 Special events and their influence on daily nutrition	2.2.1. Role of rice 2.2.2 Good and bad food 2.2.3 People, forefathers and programs’ influence on children’s diets 2.3.1 Famadihana

### Ethical considerations

On the 28th of October 2019, research authorization including ethical approval was given by the Ministry of Higher Education and Scientific Research (Ministère de l’Enseignement Supérieur et de la Recherche Scientifique (MESupReS),reference number N^o^ 098/19-MESupReS/SG/DGRS). Additionally, the chief of the Vakinankaratra region as well as the chiefs from the participating communities gave their written informed consent.

Verbal informed consent was obtained from each participant after they had been informed on various aspects of the study (e.g.study purpose, confidentiality of information, the right to refuse or stop at any point of the interview or group discussion, the voluntary nature of participation,the recording of the conversation). After completing the data collection phase, the research team went back to all locations in December. During a short meeting with the mayor’s deputy,the most important (mainly quantitative) study results were shared.

## Results

The quantitative results are presented first, in order to get an overview of WHO infant and young child feeding indicators. They are complemented with the specific topic-related qualitative findings. The remaining qualitative results are presented in the ensuing sections. They include the findings from all three used methods, as the results concurred revealed no contradictions. However, only the relevant results of the transect walks are presented in this paper. The results are presented according to the structure of the overarching themes presented in Table 4.

### WHO infant and young child feeding indicators

#### Minimum dietary diversity

**Fig. 2:**
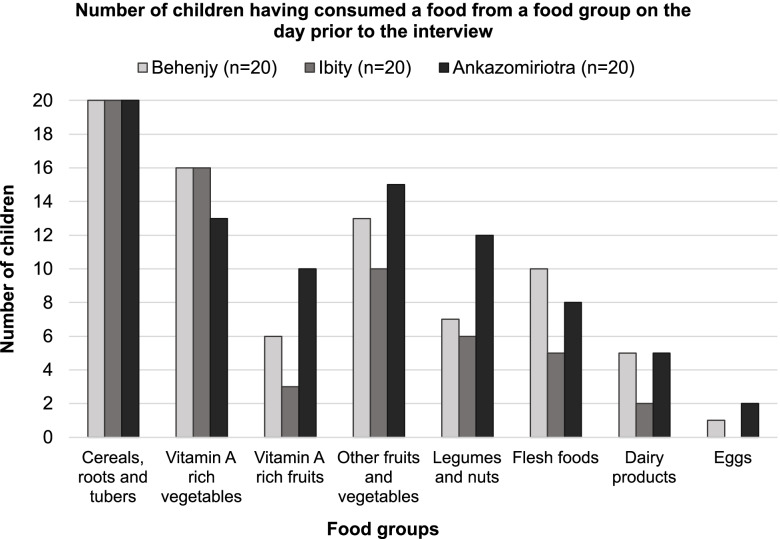
Consumed food groups by community. Results included all study children. Twenty is the maximum number of children selected in each community and indicates 100%

All included children across the three communities had received at least one kind of food from the first food group *Cereals, Roots & Tubers* the day before the interview (Fig. 2). No other food group had been consumed by all children. While around 2/3 of the children had eaten vitamin A rich vegetables, the number was lower for other fruits and vegetables. The number was lowest for animal-derived products. Ankazomiriotra had the highest number of children consuming corresponding foods for most food groups. Only flesh food was consumed more often by children in Behenjy. Children in Ibity received the least varied diet.

On average, 45% of the children achieved MDD. However, there were considerable differences between communities and age groups. In Ibity, only five out of twenty children reached MDD. 17% of children aged 24–59 months achieved MDD, compared to 38% of 6–23-month-olds. In Ankazomiriotra on the other hand, 71% of the children between 24 and 59 months and every second child below the age of two achieved this indicator. In Behenjy, 45% children were assessed to have attained MDD and no notable difference between the age groups was found (Table 5).

**Table 5:** Infant and young child feeding indicators

Indicator (WHO 2010a)	Age group	Behenjy	Ibity	Ankazomiriotra	All
	6–23 months	***n =*** 9	***n =*** 8	*n =* 6	*n =* 23
	> 23 months	***n =*** 11	***n =*** 12	***n =*** 14	***n =*** 37
**Minimum meal frequency**	6–23 months	8 (88.9%)	8 (100%)	6 (100%)	22 (95.7%)
	> 23 months	7 (63.7%)	9 (75%)	9 (64.3%)	25 (67.6%)
	All	15 (75%)	17 (85%)	15 (75%)	47 (78.3%)
**Minimum dietary diversity**	6–23 months	4 (44.4%)	3 (37.5%)	3 (50%)	10 (43.5%)
	> 23 months	5 (45.5%)	2 (16.7%)	10 (71.4%)	17 (45.9%)
	All	9 (45%)	5 (25%)	13 (65%)	27 (45%)
**Minimum acceptable diet**	6–23 months	4 (44.4%)	3 (37.5%)	3 (50%)	10 (43.5%)
	> 23 months	3 (27.3%)	2 (16.7%)	9 (64.3%)	14 (37.9%)
	All	7 (35%)	5 (25%)	12 (60%)	24 (40%)
**Consumption of iron-rich or iron-fortified food**	6–23 months	6 (66.7%)	4 (50%)	2 (33.3%)	12 (52.2%)
	> 23 months	3 (27.3%)	1 (8.3%)	6 (42.9%)	10 (27%)
	All	9 (45%)	5 (25%)	8 (40%)	22 (36.7%)

In all three communities, data from twenty children below the age of five were collected.

#### Minimum meal frequency

Most infants had received meals, snacks or breastmilk sufficiently frequently the day before the interview (96%). In all three communities, fewer children from the age group > 23 months reached MMF (68%). Usually, children receive at least three main meals. In Ibity snacks are not usual, and in Ankazomiriotra and Behenjy several children were reported to receive one snack per day when it is financially possible. Common snacks are fruit, bread or biscuits, whereby the term “bread” mostly refers to a fried ball made of rice flour and sugar.

All but one child below the age of two were breastfed. The importance of breastfeeding came up in the interviews and focus groups. In all three communities, most women mentioned exclusively breastfeeding their babies for the first 6 months and then introducing rice water as a complementary food: *“We breastfed them until they are six months and after that we start to give like water of rice [like a soup of rice, very soft rice].” (IBI-FG-02).* Toddlers’ diets do not considerably differ from adults’ diets. Exceptions for young children regarding swallowing or digestion difficulties are described further below. Children eat the same as grown-ups after the age of one, or latest at 4 years old.

#### Minimum acceptable diet

A minimum acceptable diet (MAD) was achieved by 60% of children in Ankazomiriotra, 35% in Behenjy and 25% in Ibity.

#### Iron-rich or Iron-fortified food

In Behenjy, 45% of children (more in the younger age group) consumed iron-rich foods, whilst in Ankazomiriotra 40% and in Ibity 25% had meat, fish or sausage. None of the children had received any iron-fortified products the day before the interview.

### Agricultural practices, consumption of homegrown produce & food procurement

In the transect walks and interviews it became clear that in the study region, almost everyone has access to an agricultural plot. Some own only a small field. Most interviewees grow between two and eight different crops. Crops which are produced throughout the year are listed separately for each community in Table 6. Crop diversity varies between the three communities and is lowest in Ibity. Agricultural activity is highest in November, as the rainy season starts. It falls in the lean season, as the main harvest time only starts in March/April.

**Table 6:** Commonly produced crops & fruits per community

Behenjy	Ibity	Ankazomiriotra
Cassava	Maize	Rice
Beans	Potatoes	Maize
Maize	Beans	Cassava
Rice	Rice	Peanuts
Sweet Potatoes	Cassava	Round Beans
Peanuts	Sweet Potatoes	Potatoes
Potatoes	Peanuts	Tomatoes
Green Leaves	Green Leaves	Beans
Taro	Soya	Pumpkin
Courgette	Taro	Green Leaves
Tomatoes	Green Beans	Cucumbers
Round Beans	Carrots	Onion
Pumpkin	Tomatoes	Chili
Carrots		Courgette
Lettuce		Sugarcane
Bananas	Kaki	Mango
Peaches	Oranges	Banana
Kaki	Mulberry	Pibasy (Japanese Medlar)
Oranges		Oranges
Pineapples		Pineapples
Guava		Red Bananas
Pibasy (Japanese Medlar)		Avocado
Lychee		
Mango		
Avocado		

The decision to sell rather than consume produce depends on three main reasons. The first reason for selling harvest is the family’s need for money. A further reason occurs when the harvest exceeds the family’s needs. Lastly, if the product is perishable and cannot be stored (e.g. vegetables or potatoes), it is sold. Money is often needed to buy more rice, meat, or essentials for daily use such as clothes, coffee, sugar, oil, petrol, or salt. Staples are also sold to pay for school fees, field workers or family emergencies. Peanuts and beans are considered especially valuable commodities in all three communities. Rice on the other hand is rarely sold, as it is considered a crucial staple food. As one participant put it: “*It’s the basis of our food, there is no food that can replace rice.” (BEH-FG-01).* Whilst fruits are usually consumed directly (mostly by children), beans, rice and dried cassava are stored. Stocks are usually used for feeding the family and the animals, and as seeds for the next planting season. However, in most families the harvest does not provide enough food and feed for the entire year. Foods like rice, cassava, fruits, vegetables and yoghurt or biscuits for the children need to be bought. The weekly market plays a crucial role in all three communities for this reason, as fruit, dairy products, meat and fish are purchased almost exclusively there. Fruit consumption depends highly on the season. Study participants highlighted that fruits are either not available (Ibity) or sometimes not affordable (Behenjy). Dairy products are rather expensive and consequently they are often not affordable for interviewees.

Livestock plays an important role in the study region. A few study participants own zebus. These are mainly males used for ploughing fields. Female zebus are rare. Since they produce milk, which may be consumed by the family or sold, they are even more expensive and valuable. Pigs are raised and sold in all three communities and thus serve as real-life piggy-banks. Most participants own chicken or other poultry. Generally, eggs are allowed to hatch and are rarely consumed, or only exceptionally, as highlighted by the following quote: *“No, we keep it [the eggs] to have more chickens but we don’t eat it. [ …*] *If it is accidentally broken, we eat it but... Sometimes when they [we] have many eggs, like fifteen for example, the chicken can’t cover all of them and we take off [away] maybe three or four eggs [to eat them].” (IBI-FG-01).* In Ankazomiriotra it is a little more common to eat some of the eggs: *“We eat some and we leave some to hatch. [ …] The number we eat is less than the number we sell [around 20% of the eggs are eaten].” (ANK-PI-14).* Chicken and poultry are the most commonly consumed animals. In Behenjy and Ankazomiriotra, most people who own chickens mentioned that they sell a certain amount and eat the rest. In Ibity the focus lies on selling the animals. Participants highlighted three main reasons for slaughtering chickens: 1. As a strengthening food, when feeling tired due to overwork; 2. When a family member is sick; 3. For celebrating special events. As expressed by a woman from Ibity: *“Like when we have to work a lot like this and we feel tired, when we are feeling tired, we cut… yes, we kill some chickens to eat them and [ …] when someone is ill, we have to kill it.” (IBI-PI-10).* Chicken may also be eaten if a family has enough of them. On the whole, poultry consumption varies greatly between families. While some participants eat chicken once every second week, others reported consuming poultry once every two months or less.

### Caregivers’ knowledge and perceptions of child nutrition

Study participants, especially in Behenjy and Ankazomiriotra, acquire their knowledge of child nutrition either from secondary school or from a variety of individuals and programs, such as midwives or physicians at the basic health centres or staff from nutrition centers. In Ankazomiriotra, one participant mentioned the sensitisation campaigns from the National Nutrition Office (Office National de Nutrition (ONN)) and UNICEF on TV. Others explained that they also get advice from older women in the fokontany.

In general, the study participants’ basic knowledge on child nutrition was fairly good. Some were aware of the benefit of a varied diet on child development, as emphasized by an interviewee from Behenjy: *“[ …] cheese, eggs, some fishes. Like his friends can have this, but I have not enough money to buy it. [ …] they are well for the brain growth; brain development and they have a lot of vitamins and they strengthen they give antibodies for the immune system.” (BEH-PI-12).* Meat, dairy products and fruit were the foods most often mentioned as being healthy for children across all three communities. In Behenjy and Ankazomiriotra, animal-based products as well as food with different colors were mentioned as important for child development, whereas in Ibity the focus was rather on fruits, vegetables and legumes. Additionally, in all communities, micronutrient-rich powders like *Koba Aina*, *Farilac* or Spriulina were stressed as products which participants wished they could offer more. Although participants know about healthy foods, they often cannot afford them: *“I want to give yoghurt, but I can’t afford it. It is the same for us [two other women in the focus group]. Sometimes we go on the market and we see the yoghurt and we stare at these products, but we cannot afford it.” (IBI-FG-02).*

Only a few foods were considered to be unhealthy for children. In Behenjy, legumes, cassava, cabbage, *craky chicknuts* (a snack similar to chips) and imported pasta in particular were considered difficult to digest. In Ibity, sweet potatoes and maize were mentioned as not being soft enough for the stomach when a child is sick. Eggs were mentioned as being undigestible for the stomach. In Ankazomiriotra, the age of the child played an important role. For children below the age of three there were some restrictions, such as green unripe mango, cassava, beans and avocado. However, participants also emphasized that there is no such thing as an unhealthy food, as only the amount consumed matters.

Lastly, some specific food taboos were mentioned by the study participants (Table 3). Ibity is the region with the most food taboos, whereas in Behenjy and Ankazomiriotra there were only a few. There was, however, a new rumor regarding pasta consumption and a specific birth defect. Such rumors may lead to family or fokontany-specific food taboos, examples of which are listed in Table 7. Although they do not apply to the whole community, they may have an influence on dietary diversity in certain children.

**Table 7:** Food taboos due to different beliefs

Community	Food	Quotation of taboo or belief	Source of taboo/belief
**Behenjy**	**Offal**	*“I don’t have to give him […] it’s like a rumor and I don’t know really.” (BEH-PI-01)*	Mother
	**Eggs**	*“Before when she would say “Dad” we are forbidden to give her eggs.” (BEH-PI-13)*	Mother-in-Law
**Ibity**	**Eggs**	*“P: It is forbidden to give eggs to children who doesn’t speak yet. I: […] and as soon as they speak it is okay? P: Yes, as soon as they can say “Dad”. I: And why? P: For the fear that the child will be deaf.” (IBI-FG-01)* *“The fear that they wouldn’t speak if we give [them] an egg before they can speak.” (IBI-PI-05)*	Forefathers
	**Flesh of flying birds or hedgehogs**	*“[…] if the child eats it, he will become (get), have lèpre (leprosy)” (IBI-FG-01)* *“Yes, we have some taboos. We don’t have to give children the flying bird and the hedgehog.” (IBI-PI-07)*	Forefathers > 100 years
	**Specific green leaves**	*“[…] there is a kind of green leaves we are forbidden to give that to our children.” (IBI-FG-02)*	Unknown
	**Banana**	*“[…] we are forbidden to give banana to child when they don’t have teeth yet.” (IBI-PI-11)*	Relatives
	**Liver**	*“We cannot give them until they have teeth.” (IBI-FG-02)*	Unknown
	**Sweets (bonbon)**	*“We don’t give sweetie food to child. Like sweets like bonbon.” (IBI-PI-02)*	Unknown
**Ankazo-miriotra**	**Pasta**	*“Pasta is prohibited […] It is for all the kids and even adults do not eat it. […] The reason is because since this year there was kind of disease which threatens people, the case is that a newborn baby born with a divided brain. So, people said that the cause of that is the eating of pasta. You can really see that there is a clear line which divided the skull, that’s why we call the disease “broken skull disease”. That is now the main problem that mothers face. We do not really know the cause but most of the people said that it is because of the pasta and we do not know if it is true or not. […] all the people do not want to eat pasta anymore […] There was a very cheap pasta here in the market that everyone can afford and maybe it is bad quality that’s why it’s cheaper. So, we think that because it is bad quality as cheaper, so it caused disease. So, we do not know if it is just a lie and rumor or maybe it is possible also, but all the people believe now that the cause of this disease is the pasta* “*(ANK-PI-13)* *„It doesn’t give enough energy.” (ANK-PI-16)*	*Community members*
	**Dried cassava**	*“The dried cassava is only for fifteen years old. […] There is no vitamin […] and there is more alcohol.” ANK-PI-16*	Unknown

### Special events and their influence on Children’s daily diet

In the interviews, commonly cited special yearly events included New Year’s Day, Independence Day (26th of June), Christmas and weddings, On these days, it was considered important that meals be different than usual, with more than one course if possible. Meat is the most important component, accompanied by rice, vegetables and special drinks such as fresh fruit juice or soft drinks. Thus, for celebrating these special events, extra money is needed. It is either gradually saved or obtained by working more. More agricultural products may be sold, or less goods purchased. The month in which the event takes place influences the available assets and the strategy implemented, as expressed by the following quote: *“For New Year we have to save money. But at the Independence Day we sell the food.” (ANK-PI-11)*. Some participants explained that they were used to saving money, while others stated that they had to save money for about a month to afford all the special food. In Behenjy several women mentioned that special events did not have a negative influence on their daily food intake. In Ibity and Ankazomiriotra however, most participants reported that the quantity and diversity of food consumed on the days before and after the event were reduced. In about half of these families, not only adults’ but also children’s diet was affected.

In the Vakinankaratra region, people celebrate another special event of great cultural importance: Famadihana, a ritual reburial ceremony of dead ancestors. It usually takes place every five to nine years and involves the extended family and often whole communities. As a result, several families must provide food for hundreds of people. Further money is needed for the governmental authorization fee. Moreover, in a rather new tradition, every family member should wear the same T-shirt or dress. Families also need money for transportation if the grave is far away from the place of celebration. When asked to estimate the amount of money spent for a whole Famadihana, study participants mentioned sums between 50′000 Ariary (US$13.80, half of the monthly farmer’s salary in the region) and 300′000 Ariary (US$81.52, three monthly farmer’s salaries). In some cases, a certain amount of rice had to be contributed in addition. The amounts varied between 90 and 250 kg per family. An interviewee highlighted that each guest makes a contribution. However, if you are invited back by this guest at a later point in time you are expected to give a higher contribution. Furthermore, contributions from guests are not enough to cover the expenses. As a result, the effect of Famadihana on most families’ budgets is pronounced and long-term. Although some study participants in Ankazomiriotra explained that they only need a few months to save the necessary money, most respondents start preparations more than a year before the event. As a result, many families adapt their diet, as stressed in the following quote: *“We starve a little bit [ …] every member of the family has to reduce the food.” (IBI-PI-14).* Numerous people stated that the whole family – including children –restrict their diet for quite a long period before and after Famadihana, as illustrated by the following quote: *“[ …] We must work more, and we have to reduce also what we eat. I: So, you would reduce for one year? P: Yes. I: [ …] for the whole family? Like also for the little ones? P: Yes.” (BEH-PI-04)*. This effect persists even after the event, as expressed by another participant: *“Like we have [had] some rice at home and we have [had] to spend it for the Famadihana [ …] now, as I must buy rice, I cannot afford anything else like meat. I: [ …] how long does it have this impact? [ …] P: Until the harvest of the new year. Like seven months we are like this. I: So, seven months you have to eat less diverse because of the big amount you had to spend? P: Yes.” (IBI-PI-03).*

These results suggest that special events may influence families’ diets, and the diversity thereof, far beyond the day of the celebration.

## Discussion

Our findings indicate that only about four out of ten children between 6 and 59 months achieve MAD, mainly as a result of low dietary diversity. Children’s diets mainly consist of carbohydrate rich foods, with few animal-based products, legumes and nuts. A similar pattern of high cereal and vitamin A rich vegetables and fruit consumption is found for other Malagasy regions [15] and other African countries [37–40].

In our study, less than every second child (45%) achieved MDD, in line with previous studies from other parts of the country (42%) [15], though around 10 % higher than recent published data from the Vakinankaratra region [41]. As dietary diversity is an indicator of the micronutrient density of children’s diets in Madagascar [42] – and a key to reduce stunting is to increase the consumption of nutrient-rich food [43] –a high rate of children in the study region may therefore suffer from micronutrient deficiencies, which leads to chronic malnutrition and stunting. Another study conducted by Remonja et al. (2017) in Madagascar illustrated that stunting was associated with the specific age range of 12–35 months and was possibly linked to weaning [12]. Rakotonirainy et al. (2018) investigated the WHO IYCF indicators for children of 6–59 months. They found a decrease in MAD from the age of 18 months on, even dropping to 25% in one study site [15]. Their findings are congruent with those of the present study, indicating that older children might be at a higher risk for malnutrition. The group of children aged 24–59 months was more at risk of missing a MAD than the younger one, especially when not reaching MMF due to not being breastfed anymore. These results suggest that future nutrition programs may have to focus on the diet of children after weaning.

The dietary diversity of families and children in our study region seems to be the result of several factors linked to culture, in the broad and the narrow sense. Some factors are tightly linked with economic constraints. Farming activities, especially crop diversity and livestock farming exert an influence, as does marketing agricultural produce and market access. Knowledge and perceptions of child nutrition, and to a lesser extent food taboos play a role. Lastly, cultural events, in particular Famadihana, affect intake beyond the duration of the celebration.

In rural areas in developing countries, farming practices may directly influence families’ diets. For vulnerable smallholders’ households in Malawi, crop diversity is linked to dietary diversity [44]. Kumar et al. (2015) also found a strong association between produced crop diversity and dietary diversity in Zambian children aged 6–23 months [45]. Our findings confirm these results - in Behenjy and Ankazomiriotra, more diverse crops were grown and MDD values were considerably higher than in Ibity. As this data was collected at the community level, it could indicate differences in cultivation habits between locations. These might be linked to difficult market access among other factors, as Ibity is the most remote community of the three.

The findings of the present study show that children’s consumption of animal products is low, particularly regarding dairy products and eggs. In line with our results, eggs was the least consumed food group in children below the age of two in the Vakinankaratra region [41] and in children between 6 and 59 months in other regions of Madagascar [15]. Consequently, the consumption of eggs in particular does not seem to be part of Malagasy food culture. Despite chicken being the most common livestock in the studied region, our findings indicate that almost all eggs were used for hatching, in line with the literature for other developing countries [46]. Increasing egg consumption could considerably improve children’s consumption of animal-based products and thus their dietary diversity. However, their nutritional value may not be known to caregivers, or they may not have the power to decide what is done with their eggs [47]. Ways to promote egg consumption in a culturally adapted way among caregivers in the Vakinankaratra region would need to be explored.

Regarding children’s flesh foods consumption, Rakotonirainy et al. (2018) found a higher consumption than in the present study. They showed that about 55–60% of children had eaten meat, poultry or fish the previous day, depending on the study site. In one region, this might be due to higher off-farm income opportunities, as mining operations are widespread. Interestingly, more than half of the children consumed fresh fish in the other study site [15]. In a recent study from the Vakinankaratra region, at the end of the harvest season, more than 50% of children aged 6–23 months consumed either meat, poultry or fish, with 42% small fish from rice paddies was found to be the most common food from the respective food group [41]. This indicates that fish farming may improve children’s dietary diversity in Madagascar – at least during the harvest season. As a matter of fact, projects to train people in rice-fish farming are already being carried out in the Vakinankaratra region. The potential for aquaculture is estimated to be high [48]. Future research should investigate whether keeping fish is feasible for farmers and whether it improves children’s dietary diversity.

Our study indicated that only a few female zebus were kept in the study region, and that the number of children consuming dairy products was low. Child dairy product consumption depends highly on the community’s production thereof, and remote households rely extensively on markets to provide a diverse diet for children [49]. Contrary to the other two communities, Behenjy has a daily rather than a weekly market and more opportunities for off-farm income generation. This might explain the children’s higher dairy and meat consumption. Sibhatu et al. (2015) further found that the influence of market access on dietary diversity increases for farms with a high crop diversity. People with higher cash incomes tend to purchase a bigger variety of foods at the market. These diverse foods cannot be fully gleaned from subsistence farming alone [50]. Most of our participants sold a certain number and variety of crops and bought specific foods at the market. Nevertheless, our data suggest that even though people wished to buy more diverse foods at the market, they often lacked the necessary financial means, in line with data from Rakotomanana et al. (2020), who found that low income is a barrier for optimal feeding practices [41]. Opportunities to increase household income, either through off-farm work or crop diversification, and partial selling of high-value crops, could lead to a more diversified diet for children. This however only if the additional income is used to buy nutrient-rich foods at the market [50, 51]. The association between market access and dietary diversity highly depends on the context and on factors like access to resources and nutrition education [52]. In Madagascar, dietary diversity is hardly considered when buying food at the market and preparing meals [53].

The findings of this study reveal that caretakers have some specific perceptions of child nutrition. For example, although rice is culturally the most important food and is eaten most often, only a few caregivers consider it the healthiest food. This differs from previous research, where Malagasy caregivers emphasized cereals – especially rice – as being the most nutritious food [16, 53]. Caregivers in this study avoided giving certain food to young children as it was perceived as not soft enough or not easily digestible for them, in line with evidence from other African countries [54, 55]. Furthermore, food taboos have been reported to be widespread in certain regions of Madagascar [56]. Rakotomanana et al. (2020) found such perceptions to be barriers for an optimal children’s diet in the study region [41]. In contrast, our findings show that even though some food taboos were present in all communities, their effect on children’s dietary diversity seemed to be family specific and rather limited.

In addition to food perceptions or taboos our results indicate that participants in general have a basic knowledge of child nutrition. This might be the influence of the community nutrition centers. Maternal nutrition counseling was found to contribute significantly to adequate MMF and MDD practices [57] and increases in weight and height in children 6–24 months of age in developing countries [58]. Shi and Zhang (2011) highlighted that educational intervention can improve feeding practices when they are accessible and culturally sensitive. To achieve positive changes in food practices, it is crucial that the contents of such nutritional training can feasibly be implemented in everyday life. Limiting factors such as lack of money or insufficient product availability need to be considered [59].

Last but not least, this study’s findings suggest that special cultural events and celebrations may have an influence on families’ diets and the dietary diversity thereof far beyond the day of celebration. During the event, dietary diversity seems to increase. This effect has been previously described for children in Ethiopia [60] and adolescents in Ghana [61]. In the medium term, special events rather seem to lead to financial constraints, having a negative effect on children’s dietary diversity. In South Africa, low dietary diversity scores in January may be linked to higher spending patterns and the lack of income during the festive season [62]. Nevertheless, the degree of influence that cultural events have on children’s dietary diversity depends on their size, as well as the families’ financial saving opportunities.

In this context, the most intriguing result of our study is the role of Famadihana, a large and important region-specific event. Famadihana seems to have a strong influence on dietary diversity both in children and adults. To our knowledge, this specific aspect has not been investigated before. The high economic burden, and in the case of poor households, the resulting debt has been described however, together with its great cultural importance in the Vakinankaratra region: Famadihana is used to manifest family authority, power and status. Tradition obliges invited families to donate a higher sum to the Famadiahana of a host than what they received from them at their own reburial celebration [63]. This aspect may lead to continuously high expenditures linked to this tradition. An increase in debts, especially for poor families, may result in an unbalanced, restricted diet. To our knowledge this aspect has not been described in previous research. Even though our results do not permit a quantification of this influence, a certain contribution to the high stunting prevalence may not be ruled out. The influence may be greater on children who are no longer exclusively breastfed or who are below the age of two – a critical development stage – as linear growth is especially sensitive to modifiable factors such as feeding or psychosocial care at that age [6]. The influence of Famadihana on children’s diet is certainly an aspect that should be considered in further studies.

### Strengths & Limitations

The main strength of this study is the three different qualitative approaches (transect walk, FGDs and interviews) used, which allowed for the triangulation of findings and thus a higher result validity, the mixed method design and the use of interactive pictorial tools. Visual aids have been shown to be a valuable tool to improve data collection on dietary diversity, leading to more accurate results [64].

We are aware that our results have some limitations. Convenience sampling may have resulted in a bias, as women living further away from the main street or staying mostly at home may have been excluded from the sample. Therefore, voices of active women, involved with multiple organizations and having received good informal education may be overrepresented and more traditional women, perpetuating problematic dietary beliefs and food taboos may be underrepresented. Data collection during the lean season may have influenced the results of the infant’s and young child feeding indicators. A study from Northern Ghana showed that dietary diversity may differ considerably between seasons, mainly due to the difference in consumption of fruits and vegetables [65]. The inclusion of children older than 23 months may further have implications for our results, as the WHO IYCF indicators are not designed for this age group. In the literature however, IYCF indicators with seven food groups MDD is widely used in 24–59 months old children [12, 15, 27]. The quantitative results of this study need to be interpreted with caution due to the sampling strategy and the low number of included families and children. This neither allows for representativity nor for the generalization of the findings. Careful attention must also be paid when comparing the three communities. As participants’ numbers per community were even lower, findings are rather tendencies than absolute numbers.

## Conclusions

In conclusion, the findings of this study suggest that the low dietary diversity of children under five in the Vakinankaratra region is influenced by several cultural determinants. While the implementation of crop diversity seems to be a common agricultural practice in communities, it’s adoption in the region should be further investigated, especially at the family level, due to its importance for dietary diversity. We found that caretakers dispose of a basic knowledge on child nutrition, and beliefs do not seem to influence children’s dietary diversity considerably. Further education regarding the importance of widely available animal-based products like eggs could be beneficial to children’s diets, however. Our data further suggests that the region-specific cultural ceremony of the reburial of the death Fahadimana might substantially influence dietary diversity and enhance stunting rates in children in the Vakinankaratra region. Therefore, the influence of important cultural events on dietary diversity should be part of further research.

## Data Availability

The datasets generated and analyzed during the current study are available in the Zenodo repository at the following link: https://zenodo.org/record/4719867).

## Abbreviations

DDS: Dietary diversity score
FGD: Focus Group Discussion
MAD: Minimum acceptable diet
MDD: Minimum dietary diversity
MMF: Minimum meal frequency
